# “I feel happy, I can remember things because my family is supportive of me.” Patient’s experiences of Alzheimer’s disease in Nairobi, Kenya

**DOI:** 10.1002/alz.090377

**Published:** 2025-01-03

**Authors:** Edna N Bosire, Lucy Wambui Kamau

**Affiliations:** ^1^ Aga Khan University, Nairobi Kenya; ^2^ Brain and Mind Institute, Aga Khan University, Nairobi Kenya

## Abstract

**Background:**

Alzheimer’s disease (AD) is the most common type of dementia globally and is the fifth leading cause of death and disability. About half of all people suffering from the disease are living in sub‐Saharan African Countries including Kenya. However, research on dementia has been almost exclusively focused on the Global North societies. In Kenya, little is known about patients’ lived experiences with AD. Objective: To explore patient’s experiences living with and managing Alzheimer’s disease in Kenya.

**Method:**

We employed ethnographic methods encompassing clinical and home observations and lengthy interviews among people with early‐stage Alzheimer’s disease (n = 30) in Kenya. Patients were purposively recruited from the neurology clinic at the Aga Khan University Hospital in Nairobi. We also conducted 10 home visits to understand existing social support systems and self‐management practices. Field notes and qualitative interviews were transcribed and analyzed verbatim with use of QSR NVivo 12 software.

**Result:**

Two‐thirds of the patients were aged 50 years and above, had low education levels and fully depended on close family members for support. Patients experienced challenges such as higher cost of hospital care and treatment, comorbidities (e.g., depression, diabetes and hypertension), language barrier, forgetfulness, being misunderstood and conflicts with loved ones. These challenges made managing their AD difficult. Social connectedness – which entailed: (i) Self‐connectedness – including active participation in self‐care, relaxation and having personal time; and (ii) Connectedness with others (e.g., relationships with family, friends to prevent loneliness and isolation) were found to be key on how patients managed and coped with their Alzheimer’s disease.

**Conclusion:**

People living with AD in Kenya cannot be understood without knowing about their inner lives, other people around them and the background where they belong. Alzheimer’s disease can transform people’s self‐identities but with good social support system, patients can live quality lives. There is a need to train family members and other informal caregivers to improve care and foster a sense of belonging amongst the patients. More research on Alzheimer’s disease in Kenya is needed as well as increased public awareness and community sensitization regarding dementias.

